# Синдром Видемана-Раутенштрауха. Первое описание клинического случая в Российской Федерации

**DOI:** 10.14341/probl13369

**Published:** 2023-10-08

**Authors:** А. Л. Кунгурцева, А. В. Попович, Ю. В. Тихонович, А. В. Витебская

**Affiliations:** Первый Московский государственный медицинский университет им. И.М. Сеченова (Сеченовский Университет); Первый Московский государственный медицинский университет им. И.М. Сеченова (Сеченовский Университет); Первый Московский государственный медицинский университет им. И.М. Сеченова (Сеченовский Университет); Первый Московский государственный медицинский университет им. И.М. Сеченова (Сеченовский Университет)

**Keywords:** неонатальный прогероидный синдром, синдром Видемана-Раутенштрауха, синдром преждевременного старения, РНК-полимераза III

## Abstract

Синдром Видемана-Раутенштрауха (неонатальный прогероидный синдром) — ультраорфанное заболевание из группы синдромов преждевременного старения с аутосомно-рецессивным типом наследования, ассоциированный с мутациями в генах POLR3A, POLR3B и POLR3GL, кодирующих РНК-полимеразу III. Частота заболевания в настоящее время неизвестна. Мы представляем первое в Российской Федерации клиническое описание пациента 7 лет 6 месяцев с синдромом Видемана-Раутенштрауха (компаунд-гетерозиготные мутации в гене POLR3A) с прогероидными чертами, адентией, задержкой роста (SDS роста -3,41, SDS скорости роста -2,47), дефицитом массы тела (SDS ИМТ -6,20) и генерализованной липодистрофией. В статье представлено наблюдение пациента на протяжении 1,5 года, рассмотрен мировой опыт динамического наблюдения пациентов с неонатальным прогероидным синдромом, дифференциальная диагностика, а также даны рекомендации по ведению пациентов с данным заболеванием. Учитывая отсутствие на сегодняшний день специфического лечения, пациенты наблюдаются многопрофильной командой врачей.

## Abstract (en)

Wiedemann-Rautenstrauch syndrome (neonatal progeroid syndrome) is an ultra-orphan disease from the group of premature aging syndromes with an autosomal recessive type of inheritance associated with mutations in the POLR3A, POLR3B, and POLR3GL genes encoding RNA polymerase III. The incidence of the disease is currently unknown. We present the first clinical description in Russian Federation of a patient 7 years 6 months old with Wiedemann-Rautenstrauch syndrome (compound heterozygous mutations in POLR3A gene) with progeroid features, adentia, growth retardation (height SDS -3,41, height velocity SDS -2,47), underweight (BMI SDS -6,20), and generalized lipodystrophy. The article presents the observation of the patient for 1.5 years, the world experience of dynamic follow-up of patients with neonatal progeroid syndrome, differential diagnosis, as well as recommendations for the management of patients with this syndrome. Given the lack of specific treatment to date, patients are observed by a multidisciplinary team of physicians.

## АКТУАЛЬНОСТЬ

Синдром Видемана-Раутенштрауха (неонатальный прогероидный синдром) — ультраорфанное заболевание с аутосомно-рецессивным типом наследования, ассоциированное с мутациями в генах POLR3A, POLR3B и POLR3GL, характеризующееся врожденной липодистрофией, прогероидными чертами лица и преждевременным старением. Это самый редкий из синдромов преждевременного старения и одно из самых редких заболеваний в мире (на сегодняшний день описано около 30 пациентов) [1–5].

## ОПИСАНИЕ КЛИНИЧЕСКОГО СЛУЧАЯ

Девочка от первой физиологической беременности, неродственных здоровых родителей, рожденная на 37-й неделе путем кесарева сечения. С 24-й недели беременности отмечалась выраженная задержка внутриутробного развития (ЗВУР), подтвержденная при повторных УЗИ. На 31-й неделе беременности проведено МРТ плода — выявлено шейно-окципитальное интракорпоральное менингоцеле на фоне аномалии развития краниовертебральных костных структур, вторичного расширения краниальных отделов позвоночного канала; олигоамнион (маловодие).

При рождении у пациентки зафиксированы низкие весовые показатели (при оценке с учетом срока гестации): длина — 46 см (SDS длины -0,93), масса тела — 1840 г (SDS массы тела при рождении -2,73). В первые сутки жизни при объективном осмотре обращало на себя внимание отсутствие подкожно-жировой клетчатки, гидроцефальная форма черепа с резко выраженной венозной сетью во всех отделах, микрогнатия нижней челюсти; присутствовал верхний резец верхней челюсти, самостоятельно выпавший на вторые сутки жизни.

Кариотип 46, ХХ, нормальный женский.

На третьи сутки жизни проведено МРТ головного мозга — данных за менингоэнцефалоцеле не получено, выявлены признаки наружной гидроцефалии.

Девочка была выписана из роддома на 10-е сутки с массой 1837 г.

В дальнейшем пациентка наблюдалась по месту жительства. Сохранялись выраженная задержка роста и тяжелый дефицит массы тела (рис. 1: пациентка в возрасте 1 года 3 месяцев). Отмечалась задержка моторного и психо-речевого развития. При изучении медицинской документации обращают на себя внимание частые (каждые 2–3 месяца) тяжелые обструктивные бронхиты, девочка дважды переболела двусторонней пневмонией. При проведении повторного МРТ головного мозга в возрасте 11 месяцев выявлены аномалия Арнольда-Киари 1 типа; смешанная, с преобладанием внутренней, гидроцефалия; умеренно выраженный перивентрикулярный отек головного мозга; аномалия развития затылочной кости с дефектом задних отделов большого затылочного отверстия.

**Figure:**
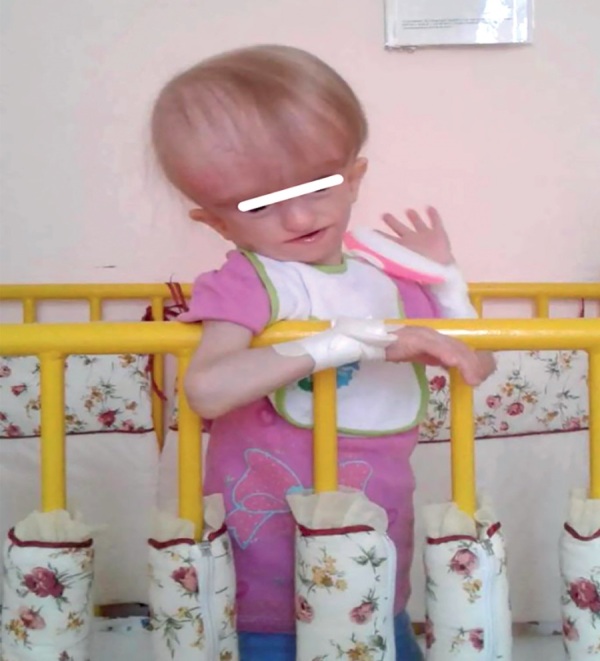
Рисунок 1. Пациентка в возрасте 1 года 3 месяцев. Figure 1. Patient aged 1 year 3 months.

В 2018 г. (3 года 5 месяцев) девочка впервые была проконсультирована генетиком, на основании объективного осмотра был заподозрен синдром Хатчинсона-Гилфорда и рекомендовано проведение молекулярно-генетического исследования.

В апреле 2022 г. (6 лет 4 месяцев) девочка была впервые госпитализирована в детское эндокринологическое отделение. При поступлении: рост — 99 см (SDS роста -3,14), скорость роста — 5,8 см/год (SDS скорости роста -1,24), вес — 10 кг, ИМТ — 10,20 кг/м² (SDS ИМТ —5,49) (рис. 2: график роста; рис.3: график ИМТ).

**Figure:**
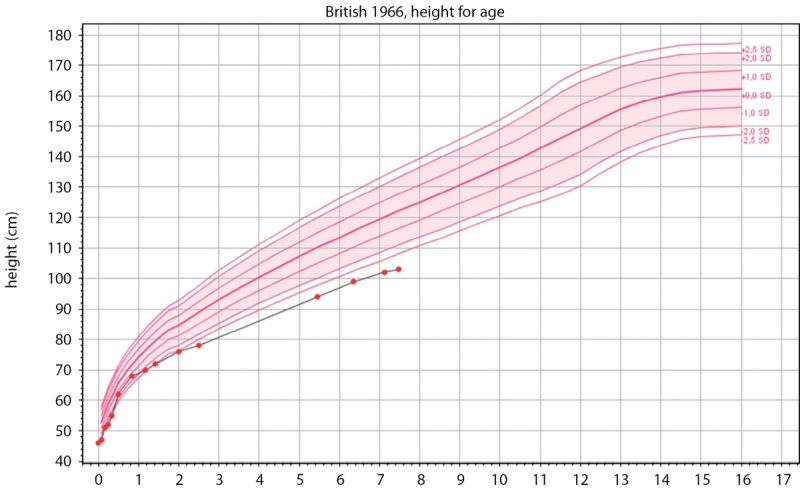
Рисунок 2. График роста. Figure 2. Growth graph.

**Figure:**
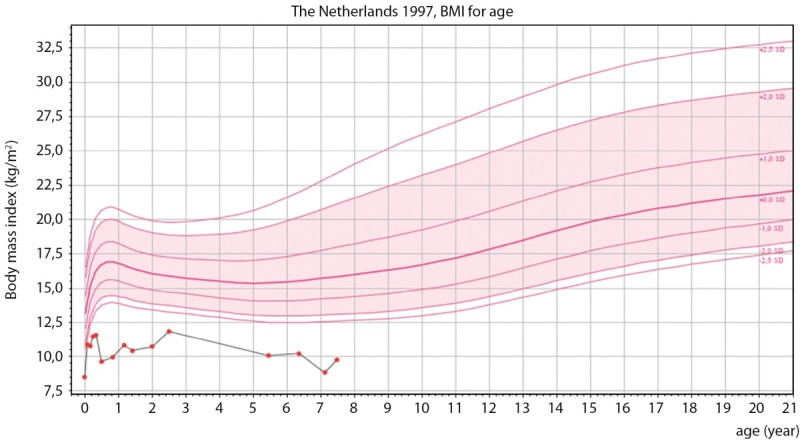
Рисунок 3. График ИМТ. Figure 3. BMI graph.

При объективном осмотре отмечалась генерализованная липодистрофия с изолированными участками перераспределения подкожно-жировой клетчатки в области шеи, наружных половых органов, копчиковой области и стоп; ограничение подвижности тазобедренных суставов, контрактуры межфаланговых суставов кистей; прогероидные черты лица (гидроцефальная форма черепа с выраженной венозной сетью, выступающие массивные лобные бугры, треугольное лицо с гипоплазией средней и нижней трети лица, редкие брови и ресницы, клювовидный нос, адентия, длинный язык), гипотрихоз волосистой части головы, поперечная ладонная складка справа. Половые органы сформированы по женскому типу, правильно. Половое развитие по Таннеру 1 (рис. 4: пациентка в возрасте 6 лет 5 месяцев; рис. 5: изолированный участок перераспределения подкожно-жировой клетчатки в копчиковой области; рис. 6: поперечная ладонная складка справа).

**Figure:**
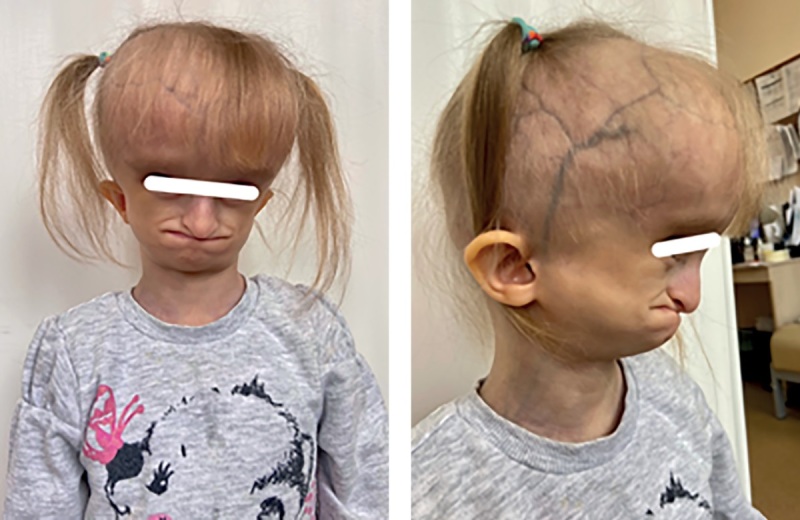
Рисунок 4. Пациентка в возрасте 6 лет 5 месяцев. Figure 4. Patient aged 6 years 5 months.

**Figure:**
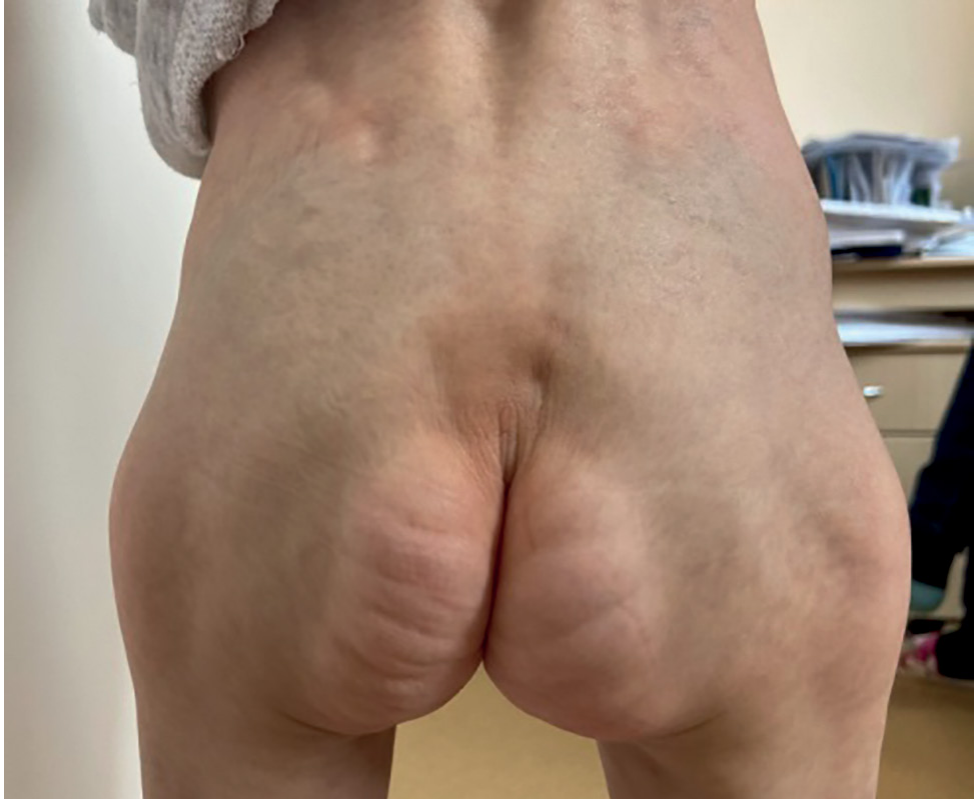
Рисунок 5. Изолированный участок перераспределения подкожно-жировой клетчатки в копчиковой области. Figure 5. Isolated area of redistribution of subcutaneous fat in the coccygeal region.

**Figure:**
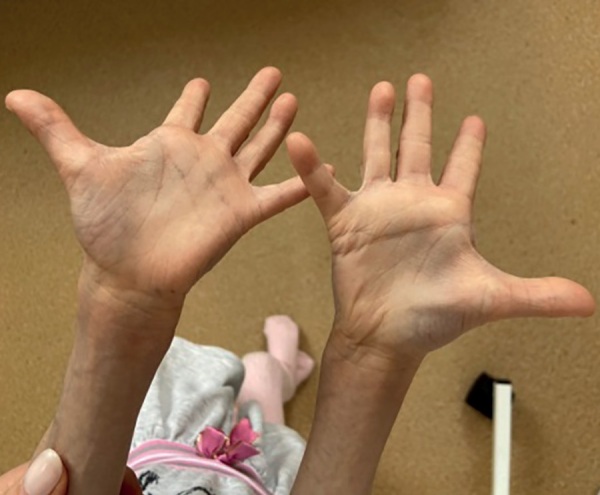
Рисунок 6. Поперечная ладонная складка справа. Figure 6. Transverse palmar fold on the right.

На основании данных анамнеза и фенотипа был диагностирован синдром Видемана-Раутенштрауха. Диагноз был подтвержден после выявления патогенных вариантов в компаунд-гетерозиготном состоянии в гене POLR3A.

В условиях отделения было проведено лабораторно-инструментальное обследование и консультации смежных специалистов. Результаты лабораторных исследований (общеклинические анализы крови, мочи и кала, биохимический анализ крови, исследование гормональных показателей) — в пределах нормальных значений. Липидный профиль — в пределах референсных значений. При проведении УЗИ органов брюшной полости, почек, щитовидной железы, ЭхоКГ, УЗ-доплерографии в дуплексном/импульсном режимах парных сосудов патологии не выявлено. По данным ЭКГ, зафиксирована умеренная синусовая тахикардия (133 удара в минуту).

При осмотре травматологом-ортопедом был выявлен диспластический тип строения позвоночника, нарушение осанки по сколиотическому типу (угроза формирования S-образного сколиоза), сгибательные контрактуры тазобедренных и коленных суставов, а также заподозрен распространенный остеопороз.

По результатам консультации невролога, подтверждены ранее диагностированные аномалия Арнольда-Киари 1 типа, spina bifida C1, дизартрия.

Пациентка также проконсультирована дерматологом (выявлен ирритантный дерматит в области кистей), офтальмологом (диагностированы ангиопатия сетчатки, периферический ангиоспазм, гиперметропия слабой степени, астигматизм), гастроэнтерологом (хронический запор).

В июне 2022 г., с целью исключения POLR3A-ассоциированной гипомиелинизующей лейкодистрофии, повторно проведено МРТ головного мозга. По результатам выявлены признаки умеренно выраженной гипотрофической наружной и симметричной внутренней гидроцефалии и врожденной остеоневральной аномалии развития кранио-цервикального сочленения.

При повторной госпитализации в июне 2023 г. (7 лет 6 месяцев) отмечалось прогрессирование задержки роста и дефицита массы тела. Рост — 103 см (SDS роста -3,41), скорость роста — 3,64 см/год (SDS скорости роста -2,47), вес — 10,35 кг, ИМТ — 9,71 кг/м² (SDS ИМТ -6,20), половое развитие по Таннеру 1 (рис. 2–3). По результатам расширенного общеклинического анализа крови, мочи и кала, биохимического анализа крови, коагулограммы клинически значимых отклонений не выявлено. В липидном профиле отмечалась тенденция к гипохолестеринемии и незначительная гипертриглицеридемия (при оценке в соответствии с возрастными нормативами): холестерин общий — 3,15–3,44 ммоль/л (N 3,26–5,30), триглицериды — 1,28–1,51 ммоль/л (N 0,40–1,24), липопротеины высокой плотности (ЛПВП) — 0,73–1,14 ммоль/л (N 0,93–1,89), липопротеины низкой плотности (ЛПНП) — 1,73–2,18 ммоль/л (N 1,76–3,63), липопротеины очень низкой плотности (ЛПОНП) — 0,58–0,69 ммоль/л (N 0,19–0,77).

Гормональные показатели — в пределах нормальных значений: тиреотропный гормон (ТТГ) — 0,63 мкМЕ/мл (N 0,4–4,0); тироксин свободный (свТ4) — 12,9 пмоль/л (N 12,5–21,5); кортизол — 202 нмоль/л (N 68,2–537); пролактин — 285 мкМЕ/мл (N 102–496); ИФР-1 — 160 нг/мл (N 78–281), SDS ИФР: +1,16. Выявлена недостаточность витамина Д (25(ОН)D — 25,9 нг/мл (норма — выше 30), несмотря на прием холекальциферола в профилактической дозе 1 000 МЕ в сутки.

Проведено исследование специфических маркеров к целиакии: титры антител к дезаминированным пептидам глиадина IgA и IgG, АТ к тканевой трансглутаминазе IgА и IgG — в пределах нормальных значений; данных за целиакию не получено.

По результатам инструментальных методов обследования (УЗИ почек, щитовидной железы, ЭКГ, ЭхоКГ, УЗ-доплерографии в дуплексном/импульсном режимах парных сосудов, ЭЭГ) клинических значимых отклонений не выявлено. На УЗИ органов брюшной полости визуализировалось умеренное количество мезентериальных лимфатических узлов максимальным размером до 14х10,6 мм.

При проведении мультиспиральной компьютерной томографии (МСКТ) органов грудной клетки, под бифуркацией между средостением и позвоночником определялось дополнительное образование размерами 41х22х3 мм, не усиливающееся при контрастировании, что расценено как лимфатический узел.

Костный возраст — 6 лет (при паспортном возрасте 7 лет 6 месяцев). С целью оценки минеральной плотности костной ткани проведена денситометрия поясничного отдела позвоночника — выявлен остеопороз (Z-критерий -3,8).

Учитывая частые бронхиты в анамнезе, проведено исследование функции внешнего дыхания (спирометрия): акустический компонент работы дыхания в нормальном частотном диапазоне, после сальбутамола — без существенной динамики, данных за активный бронхолегочный процесс нет.

По результатам обследования пациентка проконсультирована смежными специалистами. Помимо фенотипических особенностей, адентии и липодистрофии, отмечались следующие изменения.

В настоящее время девочка имеет абсолютно сохранные интеллектуальные способности (умеет хорошо считать, писать, знает наизусть песни), развитую мелкую моторику (собирает мелкий бисер, мелкую мозаику, умеет плести маленькие косички), готовится к поступлению в школу. Активно занимается с логопедом, нейропсихологом, преподавателем по вокалу (рис. 7: развитие мелкой моторики у пациентки).

**Figure:**
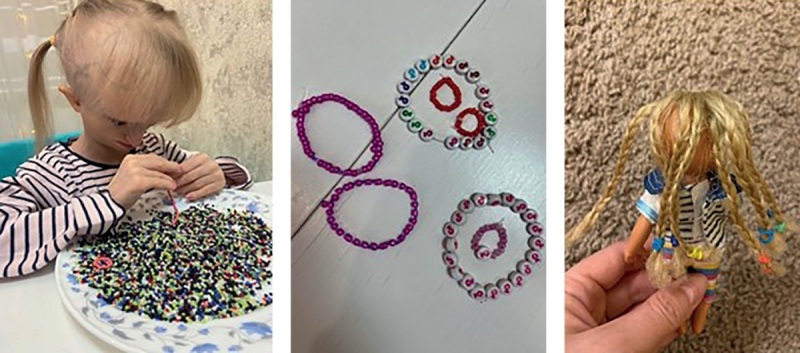
Рисунок 7. Развитие мелкой моторики у пациентки. Figure 7. Development of fine motor skills in the patient

## ОБСУЖДЕНИЕ

Первое описание пациентов с неонатальным прогероидным синдромом датируется 1977 г., когда Раутенштраух опубликовал клинические случаи двух сестер, рожденных с липодистрофией и прогероидными чертами лица [6]. Через 2 года, в 1979 г., Видеман описал двух неродственных мальчиков с врожденными прогероидными чертами лица и множественными пороками развития [7]. В 1979 г. Видеман и Раутенштраух объединили эти описания, назвав заболевание неонатальным прогероидным синдромом, а позднее это состояние стало упоминаться в литературе как синдром Видемана-Раутенштрауха (СВР) [8].

Большинство описанных клинических примеров СВР, как и в случае нашей пациентки, ассоциировано с биаллельными мутациями в гене POLR3A (локализованный на хромосоме 10q22.3), однако в последние годы появились также описания пациентов с аналогичными клиническими проявлениями при биаллельных мутациях в генах POLR3B (локализованный на хромосоме 12q23.3) и POLR3GL (локализованный на хромосоме 1q21.1) [3][9–11]. Все перечисленные гены кодируют РНК-полимеразу III, которая представляет собой ДНК-направленную РНК-полимеразу, осуществляющую транскрипцию генов, кодирующих малые РНК (рибосомальная 5S РНК, тРНК, U6 малая ядерная РНК и митохондриальная РНК-процессинговая РНК), необходимые для роста клеток, регуляции транскрипции, процессинга и трансляции РНК [3][9–13].

Первичная диагностика неонатального прогероидного синдрома возможна еще во внутриутробном периоде. В 1992 г. Castiñeyra G. и соавт. выделили ультразвуковые особенности плода с синдромом Видемана-Раутенштрауха и описали, что до срока 16–18 недель плод развивается соответственно сроку гестации, а после 18–20 недель отмечается тяжелая задержка темпов роста, особенно выраженное отставание бипариентального и брюшного размеров, маловодие [14]. У нашей пациентки тяжелая ЗВУР была диагностирована на 24-й неделе гестации при проведении планового скрининга, что согласуется с литературными данными; однако, помимо маловодия, при обследовании были выявлены также краниовертебральные деформации.

При рождении все пациенты с СВР имеют низкие росто-весовые показатели и характерный фенотип. Все исследователи обращают внимание на псевдогидроцефалию с широким передним родничком, ярко-выраженными венами скальпа, редкими и тонкими волосами; треугольную форму лица с тонкой, морщинистой и гиперпигментированной кожей (в единичных описаниях отмечались также пятна цвета «кофе с молоком»), часто пораженной атопическим дерматитом; вдавленную переносицу, клювовидный кончик носа, низко посаженные уши и короткую шею; длинные пальцы рук и ног, мышечную гипотонию. Большинство детей рождаются с генерализованной липодистрофией с единичными локализациями подкожно-жировой клетчатки в грудной, абдоминальной, ягодичной, щечной областях, однако в настоящее время описаны случаи потери подкожно-жировой клетчатки в течение первого года жизни [15–21]. Важно отметить, что у наблюдаемой нами девочки типичный фенотип, но ее особенностью является наличие не описанной у других пациентов поперечной складки на правой ладони (рис. 6).

У большинства детей с СВР, как и у нашей пациентки, при рождении имеются неонатальные резцы, однако в течение первых 4–6 месяцев жизни зубы быстро разрушаются, и дальнейшее прорезывание зубов замедляется или, чаще всего, полностью отсутствует [22].

В течение дальнейшей жизни у всех пациентов сохраняется тяжелый дефицит веса и прогрессирующая задержка роста. После 1,5–2 лет типично формирование контрактур мелких суставов, а после 6–7 лет присоединяются контрактуры больших суставов и деформации позвоночника — кифосколиоз [15][18–20]. Прогрессирование всех этих изменений мы наблюдаем у нашей пациентки.

Для многих пациентов с СВР типичны низкие уровни инсулиноподобного фактора роста-1 (ИФР-1), незначительная гиперпролактинемия. В связи с наличием генерализованной липодистрофии особое внимание уделяется нарушениям липидного обмена (гиполипидемии), есть описания дислипидемии в раннем неонатальном периоде [15][16][18]. В то же время метаболические нарушения, связанные с нарушением углеводного обмена, единичны [23]. У нашей пациентки отмечается незначительная гипохолестеринемия и гипертриглицеридемия. Уровень ИФР-1 в настоящее время в пределах возрастных значений.

При рентгенологическом обследовании обычно выявляется отставание костного возраста более чем на 2 года (в нашем случае — на 1,5 года). По результатам денситометрии поясничного отдела позвоночника у большинства пациентов отмечается значительное снижение минеральной плотности костной ткани, однако в настоящее время невозможно точно говорить об истинном остеопорозе ввиду отсутствия референсных интервалов для пациентов с прогерией и прогероидными синдромами. Несмотря на низкие показатели плотности костной ткани, на сегодняшний день предполагается, что риск переломов у данных пациентов эквивалентен тому, что отмечается у здоровых детей [16].

Дифференциальная диагностика неонатального прогероидного синдрома проводится с синдромом Хатчинсона-Гилфорда, синдромом Фонтейна и Марфано-прогероидной липодистрофией [24–30].

При рождении пациенты с СВР имеют схожие клинические проявления с больными, у которых диагностированы синдром Фонтейна и Марфано-прогероидная липодистрофия: ЗВУР, прогероидные черты лица, липодистрофии, зубные аномалии. Однако данные заболевания имеют и существенные отличительные особенности. При синдроме Фонтейна у пациентов отмечается врожденный генерализованный гипертрихоз лица и тела, пороки развития дыхательной и сердечно-сосудистой систем. Для Марфано-прогероидной липодистрофии характерны частичные проявления классического синдрома Марфана: ускоренный рост (при нарастающем дефиците веса), пороки сердечно-сосудистой системы, аномалии органов зрения и другие [31–34].

Отличительной особенностью синдрома Хатчинсона-Гилфорда, также часто называемого детской прогерией, являются нормальные росто-весовые показатели и отсутствие прогероидных черт при рождении. Первые клинические проявления отмечаются в 9–12 месяцев: выпадение волос до универсальной алопеции, потеря подкожно-жировой клетчатки, изменение фенотипа, склеродермоподобные изменения кожи. Для пациентов типичны нарушения углеводного и липидного обменов [34–35].

Учитывая наличие тяжелой задержки роста, липодистрофии, прогероидных черт лица и контрактур суставов, на первичной консультации врачом-генетиком у нашей пациентки был заподозрен более распространенный и известный синдром Хатчинсона-Гилфорда. Окончательный диагноз был установлен только по результатам молекулярно-генетического исследования.

Прогноз для жизни при СВР неблагоприятный. Средняя продолжительность жизни пациентов в большинстве случаев составляет от 7 месяцев до 2 лет, однако в литературе присутствуют описания пациентов подросткового возраста, а также одного больного 27 лет [23]. По данным Rautenstrauch T. и соавт., внешний вид с возрастом остается неизменным. Пациенты чаще всего умирают от сепсиса, вызванного аспирацирационной или бактериальной пневмонией [36].

Отсутствие подкожно-жировой клетчатки, дефицит массы тела и скелетные особенности требуют дополнительного ухода, что достаточно подробно разработано для синдрома Хатчинсона-Гилфорда и может быть предложено пациентам с СВР. Учитывая сложности в наборе веса, к привычному рациону рекомендовано добавление лечебного питания на основе смесей с гидролизированным белком. Пациенты нуждаются в длительной логопедической коррекций дизартрии с целью улучшения функции речи. Следует отдавать предпочтение одежде из хлопка или материалов, не раздражающих чувствительную тонкую кожу. При ношении обуви — обязательное использование ортопедических стелек. Рекомендовано выбирать стулья с мягким сидением/подушками. В осенне-зимнее время года следует носить одежду с дополнительным утеплением, так как пациенты с генерализованной липодистрофией быстро замерзают [37].

## ЗАКЛЮЧЕНИЕ

В настоящее время, ввиду ограниченного количества пациентов в мире и короткого периода наблюдения, не выработаны единые подходы к диагностике и коррекции осложнений заболевания [12].

В представленном клиническом случае пациентка имеет типичные для синдрома Видемана-Раутенштрауха фенотипические черты и клинические проявления. Учитывая наш и мировой опыт, пациентам с СВР может быть рекомендован следующий скрининг.

По нашему мнению, лечение и наблюдение подобных пациентов наиболее эффективно, если осуществляется многопрофильной командой врачей.

## ДОПОЛНИТЕЛЬНАЯ ИНФОРМАЦИЯ

Источники финансирования. Работа выполнена по инициативе авторов без привлечения финансирования.

Конфликт интересов. Авторы декларируют отсутствие явных и потенциальных конфликтов интересов, связанных с содержанием настоящей статьи.

Участие авторов. Кунгурцева А.Л. — вклад в концепцию и дизайн исследования, получение и анализ данных, написание статьи; Попович А.В. — получение и анализ данных; Тихонович Ю.В. — получение и анализ данных; Витебская А.В. — интерпретация результатов, внесение в рукопись существенной правки с целью повышения научной ценности статьи. Все авторы одобрили финальную версию статьи перед публикацией, выразили согласие нести ответственность за все аспекты работы, подразумевающую надлежащие изучение и решение вопросов, связанных с точностью или добросовестностью любой части работы.

Согласие пациента. Законный представитель пациента добровольно подписал информированное согласие на публикацию персональной медицинской информации в обезличенной форме в журнале «Проблемы эндокринологии».
